# Effects of intravitreal injection of siRNA against caspase-2 on retinal and optic nerve degeneration in air blast induced ocular trauma

**DOI:** 10.1038/s41598-021-96107-y

**Published:** 2021-08-19

**Authors:** Chloe N. Thomas, Alexandra Bernardo-Colón, Ella Courtie, Gareth Essex, Tonia S. Rex, Richard J. Blanch, Zubair Ahmed

**Affiliations:** 1 Neuroscience and Ophthalmology, Institute of Inflammation and Ageing, College of Medical and Dental Sciences, University of Birmingham, Birmingham, UK; 2 School of Biomedical Sciences, Institute of Clinical Sciences, College of Medical and Dental Sciences, University of Birmingham, Birmingham, UK; 3 Vanderbilt Eye Institute, Vanderbilt University Medical Center, Nashville, TN USA; 4 Ophthalmology Department, University Hospitals Birmingham NHS Foundation Trust, Birmingham, UK; 5 Department of Ophthalmology and Visual Sciences, Vanderbilt University School of Medicine, Nashville, TN USA; 6 Academic Department of Military Surgery and Trauma, Royal Centre for Defence Medicine, Birmingham, UK; 7 Centre for Trauma Sciences Research, University of Birmingham, Birmingham, B15 2TT UK

**Keywords:** Cell death in the nervous system, Cellular neuroscience, Molecular neuroscience, Visual system

## Abstract

Ocular repeated air blast injuries occur from low overpressure blast wave exposure, which are often repeated and in quick succession. We have shown that caspase-2 caused the death of retinal ganglion cells (RGC) after blunt ocular trauma. Here, we investigated if caspase-2 also mediates RGC apoptosis in a mouse model of air blast induced indirect traumatic optic neuropathy (b-ITON). C57BL/6 mice were exposed to repeated blasts of overpressure air (3 × 2 × 15 psi) and intravitreal injections of siRNA against caspase-2 (siCASP2) or against a control enhanced green fluorescent protein (siEGFP) at either 5 h after the first 2 × 15 psi (“post-blast”) or 48 h before the first blast exposure (“pre-blast”) and repeated every 7 days. RGC counts were unaffected by the b-ITON or intravitreal injections, despite increased degenerating ON axons, even in siCASP2 “post-blast” injection groups. Degenerating ON axons remained at sham levels after b-ITON and intravitreal siCASP2 “pre-blast” injections, but with less degenerating axons in siCASP2 compared to siEGFP-treated eyes. Intravitreal injections “post-blast” caused greater vitreous inflammation, potentiated by siCASP2, with less in “pre-blast” injected eyes, which was abrogated by siCASP2. We conclude that intravitreal injection timing after ocular trauma induced variable retinal and ON pathology, undermining our candidate neuroprotective therapy, siCASP2.

## Introduction

Caspases are apoptotic cysteine proteases that can drive retinal ganglion cell (RGC) death in ocular disease and after injury^1^. Caspase-2 has a prominent role in apoptosis induced by various stimuli, including DNA damage, heat shock, endoplasmic reticulum stress and oxidative stress^2–7^, and also has non-apoptotic roles in cell division, genomic stability and tumour suppression^8–12^. Caspase-2 is the most evolutionarily conserved caspase^8^ and can act as both an initiator (has a caspase recruiting domain (CARD) and can induce mitochondrial outer membrane permeabilization (MOMP)^13,14^), and an executioner caspase (substrate specificity similar to caspase-3 and -7)^15^ but does not fit into either the classically described intrinsic or extrinsic apoptotic pathways.

Caspase-2 has a pivotal role in neuronal injury, including optic nerve (ON) injury, Alzheimer’s disease and spinal cord injury^1,7,16–24^. Active caspase-2 is localised to RGC following ON crush^16,20,22^, and intravitreal injections of small interfering RNA against caspase-2 (siCASP2) protected > 95% of RGC for up to 30 days^16^, or 18 weeks if administered every 8 days^23^. siCASP2 also preserved RGC in a mouse optic neuritis^25^, and glaucoma model^16^. This siCASP2 (QPI-1007) is a naked RNA duplex with chemical modifications to prevent degradation by vitreal and serum nucleases^16,26^.

The RGCs and their axons, which form the ON, are post-mitotic central nervous system (CNS)-derived neurons and do not regenerate after injury. Degeneration of RGCs or their axons can cause irreversible visual loss. Traumatic optic neuropathy (TON), which describes traumatic injury to the ON, can occur after an explosive blast injury. Explosive blast injuries are a major cause of vision loss within the military, with 5–10% of military blast injury survivors sustaining an ocular injury^27–32^. Civilians may also be exposed to blast injuries in war zones or during terrorist attacks^33^. Retinal and ON injury after ocular trauma are more prevalent after blast-related compared to non-blast-related injuries (60% vs. 2%)^27,34,35^.

An explosive blast wave can be separated into multiple stages. The initial rapid overpressure wave is responsible for primary blast injuries. This wave is followed by a blast wind, which can carry debris and shrapnel and cause blunt and penetrating secondary blast injuries^36^. Ocular blast injury occurs in ~ 9% of US war-related eye injuries^37^, however, this is likely an underestimation as the initial physical signs might be lacking and visual loss can be delayed and progressive^38^. Repeated ocular blast injury can occur due to repeated blast wave exposure, for example, during military breacher training, where soldiers can be exposed to 12 sub-threshold blasts per day and during combat operations, with an average of 13 blasts^39^.

Our well-characterised eye-directed air blast mouse model administers an air blast wave directly to the eye, with the rest of the animal protected from the blast^40–48^. We have demonstrated retinal and ON degeneration after a single 26 pounds per square inch (psi) blast-wave at 28 days post injury (dpi)^42,45^. We have further refined this model, demonstrating that when blast exposure is repeated, even at a lower pressure, there is greater ON degeneration with relative sparing of the retina^41,42^. Thus, we refer to this model as air blast-induced ITON (b-ITON). Investigation into the cell death mechanism driving this retinal and ON axonal degeneration has identified caspase-1 mediated pyroptosis as a major driver, with antioxidants preventing injury^40^. We recently showed necroptosis activation after b-ITON and highlighted that intraocular injections within hours following the blast exposure could be detrimental and cause further ocular inflammation and degeneration^48^. Others demonstrate progressive and delayed retinal degeneration between 4 and 10 months after a single 20 psi blast wave exposure^49,50^.

We have previously shown that caspase-2 drives RGC degeneration in a rat ocular blunt impact model, which can be induced by debris carried in the blast wind, tertiary effects of blast where individuals are thrown onto a hard surface, or large objects are thrown onto them, causing blunt-force trauma with some RGC functional protection after siCASP2-induced caspase-2 knockdown^17^. Therefore, we aimed to assess if siCASP2 was also neuroprotective in a mouse model of b-ITON, when administered by either pre- or post-blast intravitreal injection, but unexpectedly demonstrated inflammation related to intravitreal injection.

## Materials and methods

### Experimental design

**Figure 1:**
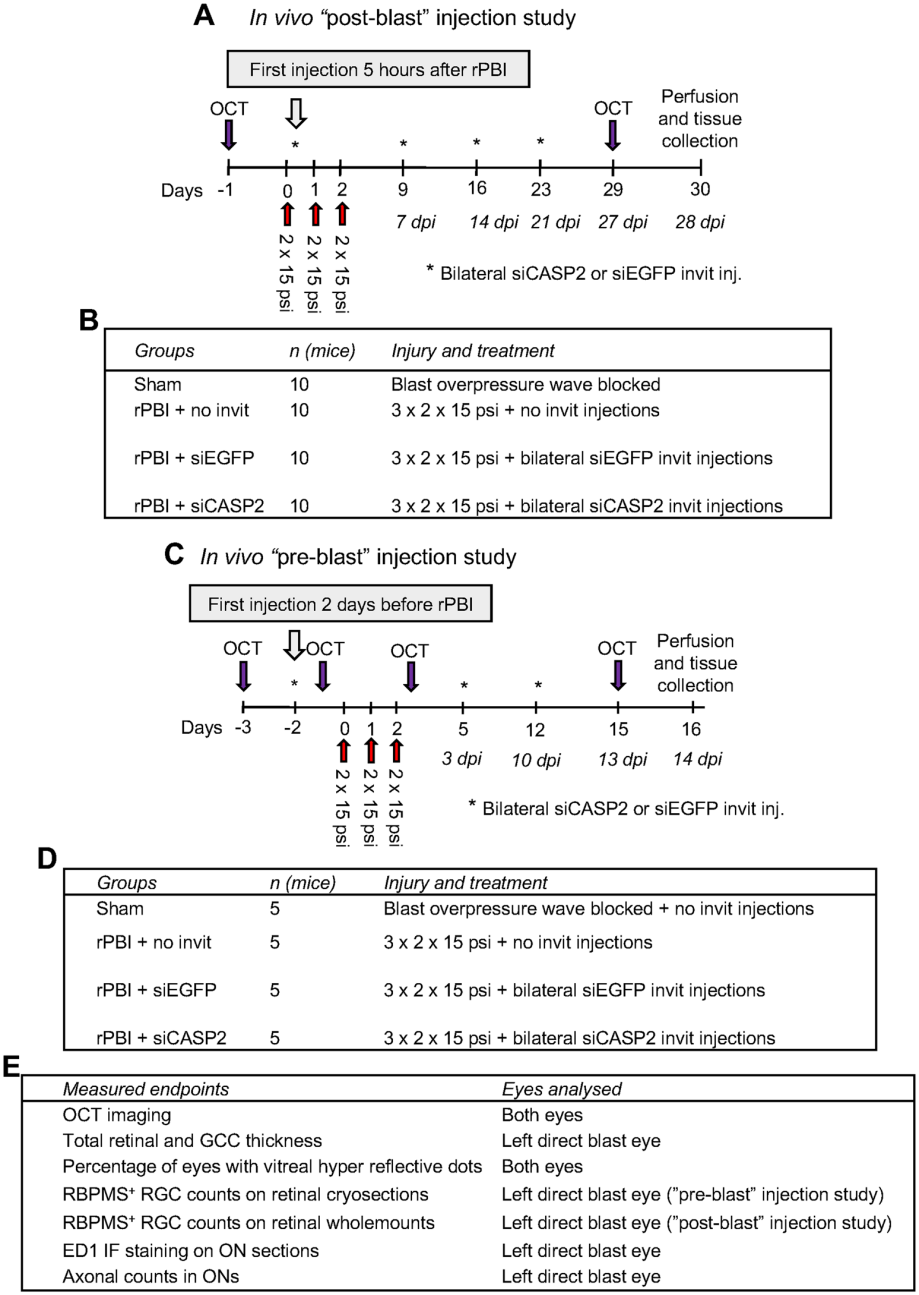
Experimental design for siCASP2 and siEGFP injection studies. (**A**) Timeline “post-blast” study. Bilateral siCASP2 or siEGFP control were intravitreally injection 5 h after the initial 2 × 15 psi blast wave, and injections repeated every 7 days until perfusion and tissue collection at 28 dpi. (**B**) Experimental groups for “post-blast” injection study. (**C**) Timeline for “pre-blast” study. Bilateral siCASP2 or siEGFP controls were intravitreally injected 48 h before the initial 2 × 15 psi blast exposure and injections repeated every 7 d until perfusion and tissue collection at 14 dpi. (**D**) Experimental groups for “pre-blast” study. (**E**) Measured endpoints and eyes analysed.

This study investigates whether caspase-2 promotes RGC death and ON axonal pathology in a b-ITON mouse model. We also considered the importance of intravitreal (invit) injection timing into an injured eye. Caspase-2 was knocked down by intravitreal injections of siCASP2 (2 μl of 1 μg/μl solution in sterile PBS) or equal concentration of siRNA against enhanced green fluorescent protein (siEGFP) as a control. In our first experiment, siCASP2 and siEGFP were intravitreally injected 5 h after the initial 2 × 15 psi blast and followed by two further blast waves, this is referred to as the “post-blast” injection study (Fig. 1A,B). Mice in this group were euthanized at 28 days post injury (dpi). We also performed injections 48 h before b-ITON, this is referred to as the “pre-blast injection” study (Fig. 1C,D). Mice in this group were euthanized at 14 dpi. Optical coherence tomography (OCT) imaging was performed bilaterally at baseline and immediately before mice were euthanized (Fig. 1E). Mice in the “post-blast” study were euthanized at 28 dpi, eyes processed for immunohistochemistry (IHC), and RGC positive for RNA-binding protein with multiple splicing (RBPMS), a specific cytoplasmic RGC marker^51,52^, were quantified on retinal whole mounts (Fig. 1E). Mice in the “pre-blast” injection study were euthanized at 14 dpi and eyes processed for immunohistochemistry (IHC) and RBPMS^+^ RGC quantified in retinal cryosections, as previously described^48^. In both groups, the far proximal ON tissue was processed for resin semi-thin cross-sections and PPD-stained intact and degenerating ON axons were quantified. The remaining ON were processed as longitudinal cryosections for IHC analysis (Fig. 1E).

### Animal care and procedures

12-week-old male C57BL/6 mice purchased from Jackson Laboratory (Bar Harbor, Maine, USA) were used in this study. Animal procedures were approved by the Institutional Animal Care and Use Committee of Vanderbilt University and conformed to the Association for Assessment and Accreditation of Laboratory Animal Care guidelines and conducted in accordance with the ARVO Statement for the Use of Animals in Ophthalmic and Vision Research. The study was carried out in compliance with the ARRIVE guidelines. Animals were randomly assigned, and the experimenters masked to the treatment and procedural conditions. All procedures and investigations were performed between 07:00 and 12:00.

### Air blast-induced indirect optic neuropathy (b-ITON) injury mouse model

Animals were anaesthetised using 3% isoflurane in 1.8 l/min of O_2_ and male 12-week-old C57BL/6 mice were exposed to a blast overpressure wave produced by a device modified to produce an air blast wave, as previously described^40–42,47,48^. Repeated air blast wave injury was chosen to more closely approximate real-world blast injury from linked mines or to mimic the multiple blast wave exposures and blunt-force injuries that occur during a single large explosive blast event. We used a 15 psi air blast, which alone does not cause pathology^42^. The average of 8 air blast tests shows that the system induces peak pressure at the location of the eye, when the mouse is in the system, at 4 ms post-trigger, stays elevated for 1 ms, and returns to baseline by 9 ms. This repeated paradigm causes greater RGC axonal degeneration compared to a single 26 psi air blast wave exposure. The left eye of mice was exposed to two 15 psi air blast waves with an interblast interval of ~ 0.5 s, repeated for 3 consecutive days for a total of 6 exposures^42^. The mouse eye was positioned 162 mm from the end of the device. Separate mice were exposed to equivalent procedures excluding the air blast wave which was blocked and verified to deliver a pressure of < 2 psi, and did not receive intravitreal injections. A pressure transducer recorded the air blast overpressure wave, which was viewed using LabVIEW software (National Instrument Austin, TX, USA). GenTeal Tears (Alcon, Novartis, Fort Worth, Texas, USA). Eye drops were applied after the air blast waves to prevent corneal dehydration from anaesthetic exposure and the mice were allowed to recover fully.

### Intravitreal injection of siCASP2 and siEGFP

Intravitreal injections were performed using a 31-gauge needle with a bevelled tip attached to a 10 μl Gastight Syringe (Hamilton, Reno, NV, USA) under inhalational anaesthetic of 2–3% isoflurane at a 45° angle 1 mm peripheral to the limbus and the lens was avoided. Unilateral b-ITON was performed and 2 µl of 1 μg/μl siCASP2 or siEGFP (provided by Quark Pharmaceuticals Inc. under a Material Transfer Agreement) as control administered by bilateral intravitreal injection. Full details of siCASP2 and all modifications to the sequence are detailed as described previously^16,26^. Briefly, siCASP2 is a naked RNA duplex with chemical modifications to prevent degradation by vitreal and serum nucleases. In the first “post-blast” injection study, siRNA injections were performed 5 h after the initial blast wave and repeated every 7 days until killing and tissue collection at 28 dpi (n = 10 per group) (Fig. 1B). In the second “pre-blast” injection study, injections were performed 48 h before the b-ITON (n = 5 per group) and repeated every 7 days until euthanasia and tissue collection at 14 dpi (Fig. 1D). Animals were perfused under terminal anaesthesia as described below.

### Optical coherence tomography (OCT) imaging, retinal thickness and vitreous haze analysis

OCT scans were performed under anaesthesia (3% isoflurane in O_2_) at 27 dpi in the post-blast group and 13 days in the pre-blast group, to construct a high resolution cross-sectional retinal image using a Bioptigen ultra-high-resolution SD-OCT system with a mouse retinal bore (Bioptigen, North Carolina, USA). Pupils were dilated using 1% tropicamide and GenTeal™ lubricant gel was used to maintain corneal clarity and prevent drying. All images were acquired with the same level of A-scan averaging (100 averages per A scan) and with the retinal position central to the image. A total of 2 B-scans were analysed per eye either side of the ON head. Whole retinal thickness and ganglion cell complex (GCC) thickness (ganglion cell layer, GCL, and inner plexiform layer, IPL) were measured in OCT images in line with the optic nerve head (ONH). Image J was used to manually segment the layers and measure the area, which was divided by the length of the retinal segment measured to calculate the layer thickness. Analysis to quantify vitreous inflammation was performed using Image J (http://rsbweb.nih.gov/ij), based on the method previously described^53^: two images either side of the ONH were analysed per eye and the pixel intensity in five regions of interest in the vitreous were measured and then displayed as a percentage of the average of retinal pigment epithelium (RPE) intensity. Results are displayed for “pre-blast” and “post-blast” studies and we have also grouped siCASP2 and siEGFP injections from the “pre-blast” and “post-blast” intravitreal injection groups and compared to sham and no invit eyes to determine if intravitreal injections cause changes in vitreal inflammation.

### Tissue preparation for IHC

Animals were euthanized by overdose of anaesthetic (Isofluorane) and intracardially perfused with 4% EM-grade paraformaldehyde (PFA; Electron Microscopy Sciences, Hatfield, Pennsylvania, USA) dissolved in phosphate buffered saline (PBS). Eyes were then cryoprotected in ascending concentrations of sucrose (10%, 20%, 30%) and embedded in optimal cutting temperature compound. Sections were cut at 15 µm-thick using a cryostat (Bright Instruments, Huntingdon, UK), collected onto SuperFrost (Fisher Scientific, Loughborough, UK) coated glass slides and stored at − 20 °C until required for IHC. Whole retinae were dissected out of the eyes and IHC staining performed in 24-well plates, as described below.

### Immunohistochemistry (IHC) in retinal frozen sections

Frozen cryosections were thawed for 20 min, washed in several changes of PBS, permeabilised and non-specific binding sites blocked by incubation in PBS containing 1% Triton-X-100 (Sigma) and 3% bovine serum albumin (BSA; Sigma). Sections were then incubated overnight at 4 °C with the appropriate primary antibody (Table 1) before washing in several changes of PBS and incubating with Alexa Fluor 488/594 or HRP-labelled secondary antibodies (Table 1) for 1 h at room temperature (RT). Finally, sections were washed in PBS and coverslips mounted in a Vectashield antifade aqueous mounting medium with 4′,6-diamidino-2-phenylindole (DAPI) nuclear stain (Vector Laboratories, Peterborough, UK). Controls, which included omission of primary antibody were included in each run and were used to set the background threshold levels prior to image capture.

**Table 1:** Primary and secondary antibodies used in this study.

Antibody	Detects	Host	Dilution	Source	Catalogue number
RBPMS	RGC marker	Rabbit	1:400 (IHC)	Millipore	ABN1362
BRN3A (14A6)	RGC-specific transcription factor	Mouse	1:200 (IHC)	Santa Cruz	SC-8429
GFAP	Activated glia	Mouse	1:200 (IHC)	Sigma	G3893
ED1	Macrophages	Rabbit	1:200 (IHC)	Abcam	AB125212
Alexa Fluor 488	Mouse IgG	Donkey	1:400 (IF)	Invitrogen, molecular probes	A21202
Alexa Fluor 594	Rabbit IgG	Donkey	1:400 (IF)	Invitrogen, molecular probes	A21207

*IHC* immunohistochemistry, *IF* immunofluorescence.

### IHC in retinal wholemounts

Retinal wholemounts were stained with appropriate primary antibodies after permeabilization in PBS containing 0.5% Triton X-100 (Sigma). To aid permeabilization, this step was first performed at − 80 °C for 15 min, followed by thawing in 0.5% Triton X-100 for 15 min at RT. Primary antibodies were diluted in PBS containing 2% Triton X-100 and 2% BSA (all from Sigma), added to wholemounts and incubated overnight at 4 °C. Wholemounts were washed in several changes of PBS, incubated with appropriate secondary antibody for 2 h at RT, washed in PBS and coverslips mounted using Vectashield aqueous mounting media containing DAPI. Primary antibodies were omitted in controls and were used to set the background threshold levels. Wholemounts were viewed under an Axioplan 2 fluorescent microscope equipped with an AxioCam HRc and running Axiovision Software (All from Zeiss, Hertfordshire, UK). An experimenter masked to the treatment conditions captured representative images from each wholemount for analysis.

### Assessment of RGC survival

The number of RBPMS^+^ immunostained RGC were quantified. In the “post-blast injection” study, RBPMS^+^ RGC were counted in the middle portion of the retinal whole mounts (Supplementary Fig. S1A) and displayed as mean RBPMS^+^ RGC per mm^2^. In the “pre-blast injection” study, RBPMS^+^ RGC in the GCL were counted on retinal cryosections in line with the ONH in pre-determined areas (central, middle and peripheral) retina (Supplementary Fig. S1B) and displayed as mean RBPMS^+^ RGC/mm of retina, as previously described^48^. Four images were analysed from two cryosections per animal.

### Resin-embedded ON and p-phenylenediamine (PPD)-staining

The ONs were collected and fixed in PBS containing 4% electron microscopy (EM)-grade PFA (Electron Microscopy Sciences, Hatfield, PA, USA), followed by 2% glutaraldehyde in 0.1 M cacodylate buffer for 2 h on ice, then post fixed in 2% osmium tetroxide in cacodylate buffer and dehydrated in ascending concentrations of 200-proof ethanol (50%, 70%, 95% and 100%) and further dehydrated in propylene oxide, as previously described^44,45,53,54^. ON tissues were embedded in Epon epoxy resin (Electron Microscopy Sciences, Hatfield, PA, USA) and 1 μm thick semi-thin sections cut using a Leica Microtome and stained with 1% PPD (Sigma-Aldrich Corp., St. Louis, MO, USA) and 1% toluidine blue (Fisher, Watham, Massachusetts, USA). Slides were imaged using 100× oil immersion objective on an AxioPlan microscope (Carl Zeiss, Hertfordshire, UK) and manually compiled using Adobe Photoshop software (Adobe Systems Inc., San Jose, CA, USA).

### Counting of intact and degenerative ON axonal profiles

**Figure 2:**
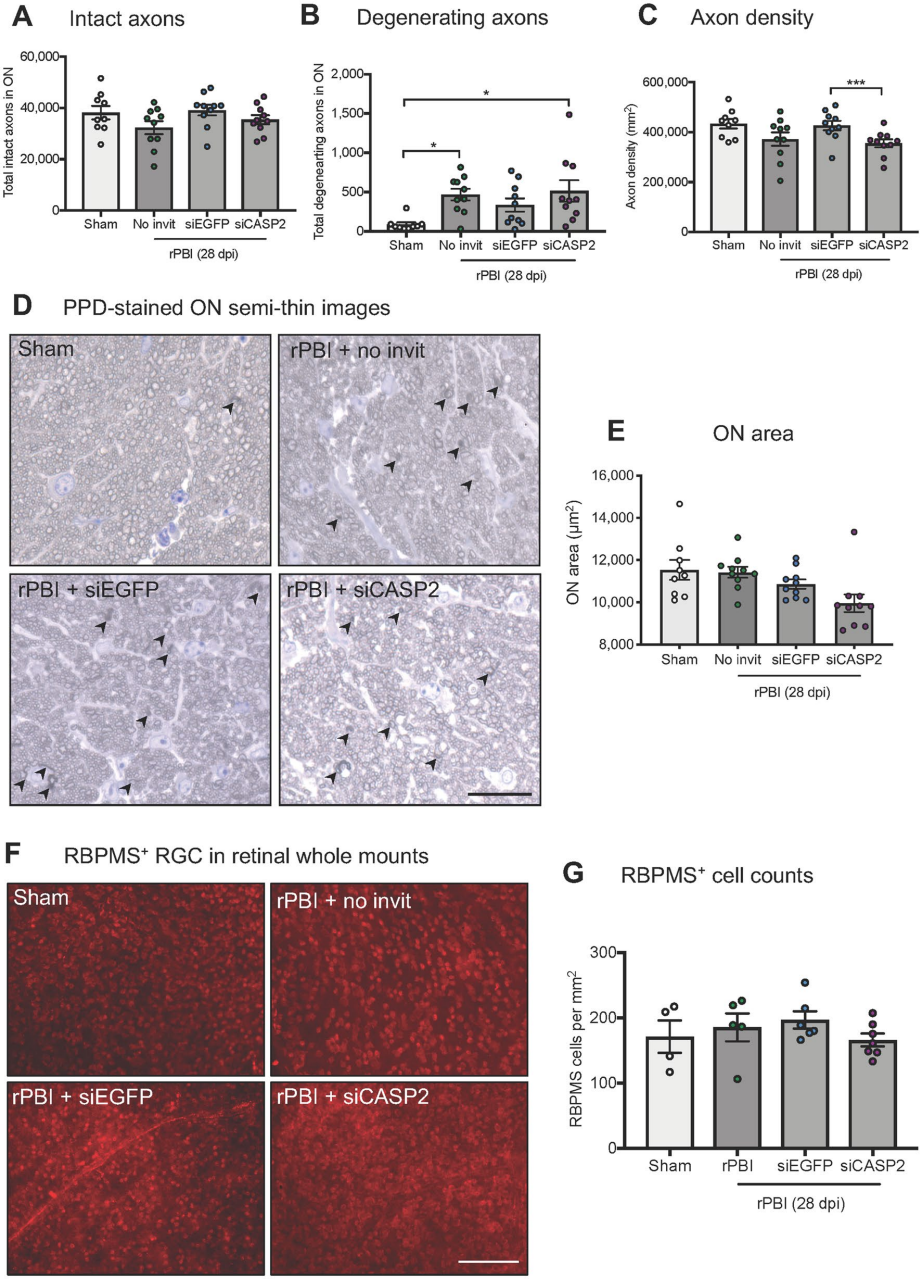
The number of ON axons and RBPMS^+^ RGC after b-ITON and “pre-blast” siCASP2 injection. (**A**) There were no differences in total intact ON axon counts between any groups (P = 0.156, ANOVA). (**B**) The total number of degenerating ON axons in the whole ON increased after b-ITON compared to sham (P = 0.0124, ANOVA; P = 0.0317, post-hoc Tukey) and remained higher than sham in b-ITON eyes injected with siCASP2 or siEGFP injections (P = 0.5112, post-hoc Tukey (siEGFP vs siCASP2)). (**C**) There were differences in ON axonal density (P = 0.0241, ANOVA), with a decreased axonal density in b-ITON eyes treated with siCASP2 compared to siEGFP control (P = 0.0097, Tukey). (**D**) Representative PPD and toluidine blue stained resin semi-thin ON sections, with degenerating axons (arrowheads). (**E**) The ON area was significantly different between groups, with eyes injected with siCASP2 displaying smaller ON area compared to sham and b-ITON alone (P = 0.0125, ANOVA; P = 0.0182 (siCASP2 vs sham) and P = 0.0263 (siCASP2 vs b-ITON), post-hoc Tukey). (**F**) Representative RBPMS^+^ immunostained RGC on retinal wholemounts. (**G**) There were no differences in RBPMS^+^ RGC counts across all groups (P = 0.505, ANOVA). Scale bars in (**D**) = 20 μm and (**E**) = 100 μm. Error bars represent mean ± SEM.

The total ON area and the number of ON axons with intact and degenerative profiles were quantified, as described by us previously^48^. Briefly, the circumference of the ON was measured and the total ON area calculated using Image J (NIH, Maryland, Bethesda, USA). The numbers of intact and degenerating (arrows Fig. 2D) ON axons were counted manually by assessing the morphological characteristics of PPD stained axons. Intact axons had uniform myelin whereas degeneration axons had unravelling or collapsed myelin. ON axons in 9 boxes in the Image J Counting Grid plug in were analysed and the total number of axons in the whole ON extrapolated.

### Statistical analysis

Statistical analysis was carried out using SPSS version 26 (IBM Corp, Armonk, NY, USA) and GraphPad Prism version 7.00 (GraphPad Software, La Jolla, CA, USA). The data were tested for normality using the Shapiro–Wilk test. Normally distributed data without linkage were analysed using one-way ANOVA and post-hoc Tukey tests with P values corrected for multiple comparisons (RBPMS^+^ RGC quantification, ON axon counts, ED1^+^ cells in ON, retinal thickness in OCT scans). Data including measurements from two eyes of each animal (vitreal intensity in OCT scans) were modelled using generalised estimating equations (GEE; normal distribution with identity link function and independent correlation matrix). To construct a model to fit the data, all available factors were included in the initial model with 2-way interaction terms and then terms with P > 0.05 were serially removed from the model, starting with the least significant and interaction terms. In experiments where multiple comparisons were made, the P values were corrected with Bonferroni correction. Data is reported as mean ± standard error of the mean (SEM). Sample size was based on previous studies demonstrating that n = 5 animals per group detected treatment effects on axon counts^40,48^. No animals were excluded or euthanized due to reaching humane end points before study completion.

## Results

### The number of degenerative ON axons after b-ITON was not affected by “post-blast” siCASP2 intravitreal injections

We first determined the effects of caspase-2 knockdown in b-ITON on RGC degeneration, ON axonal pathology and vitreal inflammation. To do this, we performed “post-blast” intravitreal injections of our well-characterised siCASP2^16,17,22,23^ or a control siEGFP, and then assessed ON axonal morphology, quantified the number of degenerating and intact axons as well as axon density on PPD-stained semi-thin proximal ON cross-sections (Fig. 2A–D). There was no difference in total axonal numbers between sham and b-ITON without injections at 28 dpi (38,193 ± 2639 vs 32,351 ± 2541 total axons in ON) or after intravitreal siCASP2 or siEGFP injections (35,451 ± 1770 vs 39,190 ± 2082 total axons in ON) (P = 0.156, ANOVA). However, total axon numbers in all groups were lower than expected when compared to known C57BL/6 J mouse ON axon numbers of 45,000–55,000^55–57^. We did observe an increase in degenerating axons in the b-ITON without intravitreal injections, compared to sham controls (467 ± 75.3 vs 86.67 ± 26.87; P = 0.0124, ANOVA; P = 0.0317, post-hoc Tukey). The number of degenerating ON axons was also elevated compared to sham eyes in those receiving b-ITON and intravitreal siCASP2 or siEGFP injections, but with no difference between these two groups (514 ± 136 and 335.5 ± 84.34, respectively; P = 0.5112). Interestingly, while we did not detect a statistically significant difference between total axon density in the sham and no injection b-ITON groups, there was a difference between the siCASP2-injected eyes compared to siEGFP-injected eyes (355,354 ± 15,784 vs 426,567 ± 18,893 axons per mm^2^, respectively; P = 0.0241, ANOVA). The ON area was significantly different between groups, with eyes injected with siCASP2 displaying smaller ONs compared to sham and b-ITON alone (P = 0.0125, ANOVA; P = 0.0182 siCASP2 vs sham; and P = 0.0263 siCASP2 vs b-ITON alone, post-hoc Tukey; Fig. 2E).

### The number of RBPMS^+^ RGC did not decrease up to 28 dpi in b-ITON or with “post-blast” intravitreal injections

**Figure 3:**
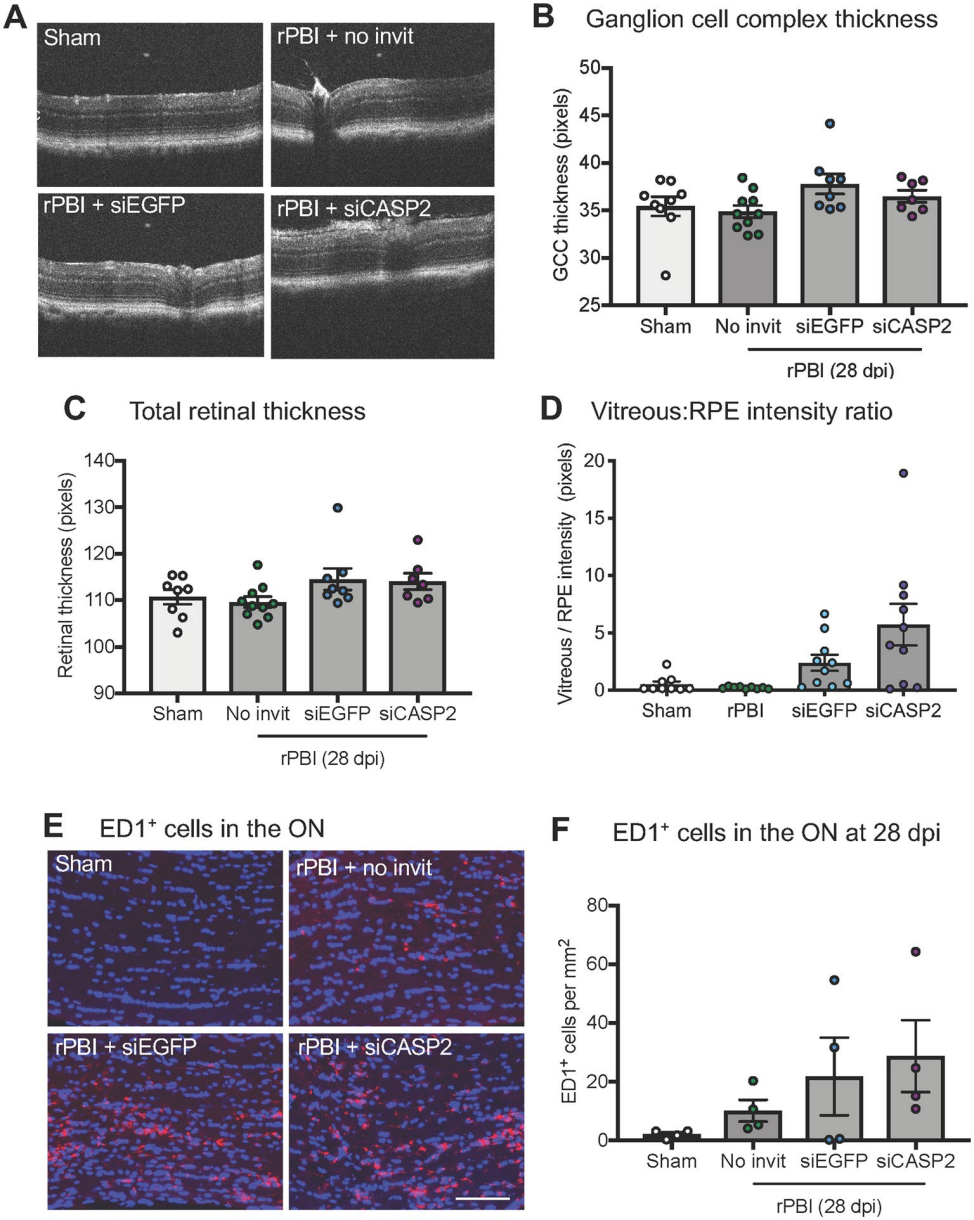
Increased vitreal intensity after “post-blast” siCASP2 intravitreal injections, but no changes in retinal thickness and ED1^+^ cells in the ON after b-ITON. (**A**) Representative OCT scans. (**B**) There were no difference in GCC thickness between any groups (P = 0.121, ANOVA). (**C**) Vitreous intensity normalised to RPE intensity increased after b-ITON with siEGFP and siCASP2 intravitreal injections compared to b-ITON with no injections (P = 0.001, GEE) and in eyes receiving siCASP2 injections compared to siEGFP injections (P = 0.002, post-hoc Tukey). (**D**) There were no differences in total retinal thickness between any groups (P = 0.1012, ANOVA). (**E**) Representative ON immunofluorescent images stained for DAPI (blue) and ED1 (red). (**F**) ED1^+^ cells infiltrate the ON after b-ITON and with “post-blast” injections, although ED1^+^ cell counts did not reach significance (P = 0.23, ANOVA). Scale bar for (**E**) = 100 μm. Error bars represent mean ± SEM.

Next, we assessed RGC degeneration by counting RBPMS^+^ RGC on retinal whole mounts in sham, b-ITON and intravitreal injections of siCASP2 and siEGFP after b-ITON. There were no changes detected in the number of RBPMS^+^ RGC at 28 dpi (P = 0.505, ANOVA; Fig. 2F,G), suggesting that RGC might not degenerate up to 28 dpi with no ability to detect siCASP2 neuroprotective effects if any at this time. For example, the number of RBPMS^+^ RGC per mm^2^ was 406.4 ± 58.85 in sham, 440.2 ± 50.59 in b-ITON injured eyes with no intravitreal injections, 467.10 ± 31.41 in b-ITON with siEGFP injections and 394.4 ± 23.54 in b-ITON with siCASP2 injections (P = 0.505, ANOVA). GCC thickness on OCT was also unaffected by b-ITON or by b-ITON followed by siCASP2 or siEGFP injections (P = 0.101, ANOVA; Fig. 3A,B). Furthermore, there were no changes in total retinal thickness at 28 dpi (Fig. 3C; P = 0.121, ANOVA).

### Intravitreal injections caused vitreal inflammation but had no effect on retinal thickness

To assess vitreal inflammation, we performed posterior segment OCT scans (Fig. 3A) and measured the vitreal intensity and normalised this against RPE intensity. There was no evidence of an effect on vitreous intensity of b-ITON without intravitreal injections compared to sham, but there was a strong effect of giving intravitreal injections after b-ITON compared to b-ITON eyes receiving no injections (P < 0.001 for b-ITON vs b-ITON + siCASP2 and b-ITON + siEGFP grouped, GEE). Moreover, there was a greater vitreous intensity after siCASP2 intravitreal injections, 5.72 ± 1.78% intensity (vitreous/RPE) compared to eyes receiving siEGFP injections, 2.41 ± 0.70% intensity (vitreous/RPE) (Fig. 3D; P = 0.001, GEE). This suggests an inflammatory effect of intravitreal injections and potentially an effect of caspase-2 knockdown in this model, although siCASP2 itself does not provoke an inflammatory or interferon response per se^16^.

### Infiltration of ED1^+^ cells in the ON by 28 dpi

To assess infiltrating inflammatory cells in the ON after b-ITON, we counted ED1^+^ cells in longitudinal ON cryosections (Fig. 3E,F). ED1 is a widely used monoclonal antibody clone which is directed against CD68 and is used to identify macrophages, monocytes and activated microglia in rat tissues. While there appeared to be more ED1^+^ cells in the ON after b-ITON, the numbers did not reach statistical significance in any group. For example, there were 2.09 ± 0.68 ED1^+^ cells per mm^2^ were present in sham ONs, we detected 10.11 ± 3.69, 21.79 ± 13.21, and 28.73 ± 12.21 ED1^+^ cells per mm^2^ in the uninjected b-ITON, “post-blast” siEGFP and siCASP2 injected b-ITON ONs, respectively (P = 0.2255, ANOVA).

These results demonstrate that markers of ON and retinal inflammation, including ED1^+^ macrophage-infiltration and vitreous haze increased in concert after b-ITON followed by intravitreal injection, with a possible additive effect when the intravitreal injection of siCASP2 knocked down caspase-2 levels.

### ON axonal degeneration and RBPMS^+^ RGC after “pre-blast” siCASP2 and siEGFP intravitreal injections

The study detailed thus far was performed with siCASP2 or siEGFP intravitreal injections at 5 h after the first 2 × 15 psi blast exposure, into a potentially vulnerable and traumatically inflamed eye, which we have demonstrated caused vitreous and retinal inflammation. To determine if intraocular injections at this time point were potentially detrimental to the eye, we next performed a further study in which we injected siRNA at 48 h before the b-ITON, “pre-blast” injection. Pre-injury siCASP2 intravitreal injection is unlikely to be clinically translatable, however, we aimed to derive information on pre-injury caspase-2 knockdown and the mechanistic role of caspase-2 in the b-ITON model. Therefore, we injected siCASP2 and siEGFP at 48 h before b-ITON and continued the study for 14 dpi (n = 5 mice per group).

**Figure 4:**
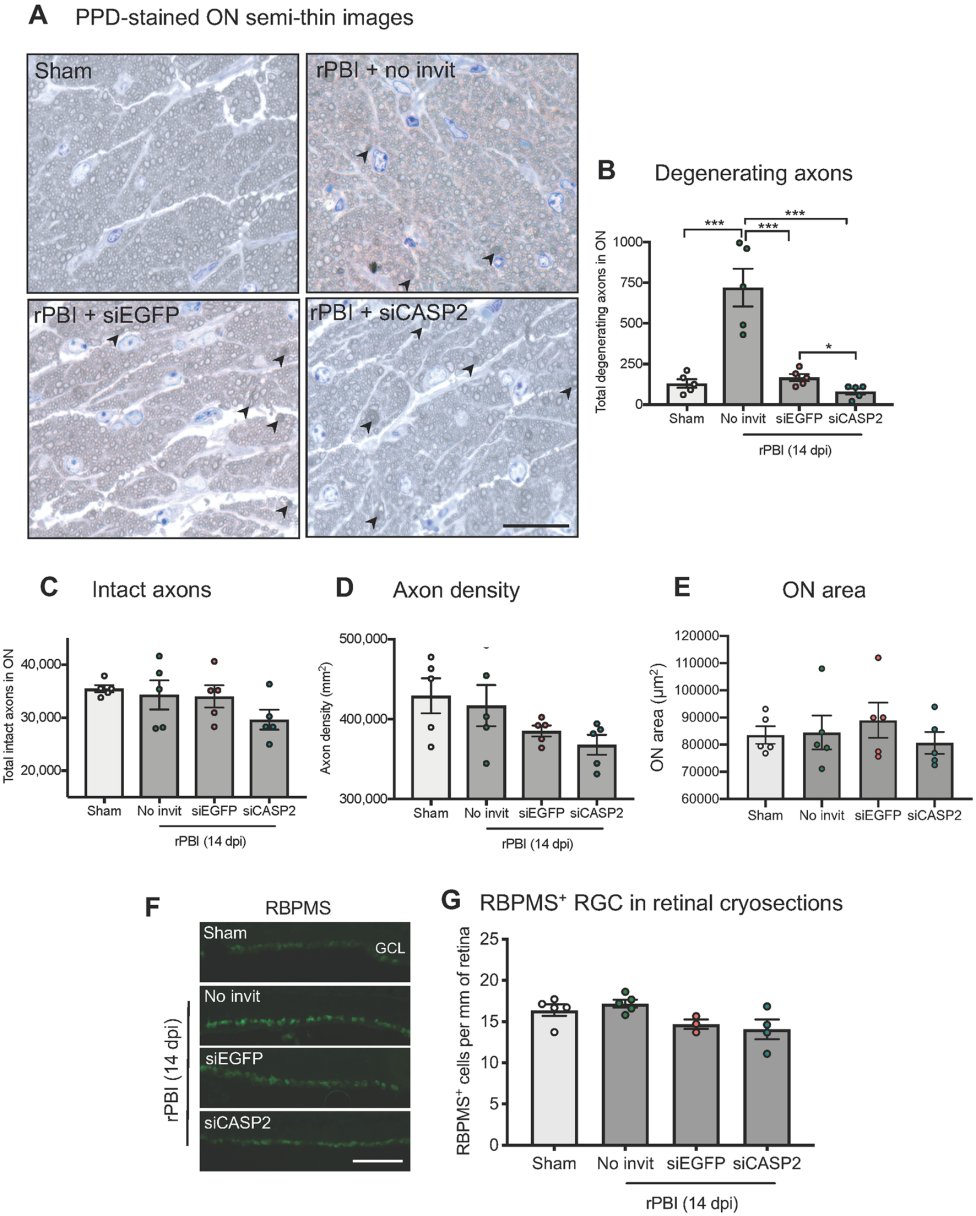
Axonal counts in PPD-stained ON semi-thin sections and RBPMS^+^ RGC in retinal cryosections in the “pre-blast” injection study. (**A**) There were increased total degenerating axons in the ON after b-ITON compared to sham (P < 0.0001, ANOVA) but not in b-ITON eyes intravitreally injected with siCASP2 or siEGFP control (P < 0.001 for both siCASP2 and siCASP2 compared to b-ITON, post-hoc Tukey). The number of degenerating axons was lower in siCASP2 treated eyes compared to siEGFP controls, indicating a potential axon-protective effect of caspase-2 knockdown (P = 0.0170, post hoc Tukey). (**B**) There were no differences in intact axons between any groups (P = 0.214, ANOVA). (**C**) There was weak evidence of differences in ON axon density between sham and b-ITON groups (P = 0.1099, ANOVA). (**D**) Representative PPD and toluidine blue-stained resin semi-thin ON sections, with degenerating axons (arrowheads). (**E**) There were no differences in ON area between groups (P = 0.7244, ANOVA). (**F**) Representative IHC images showing RBPMS^+^ RGC in the GCL on retinal cryosections. (**G**) There was weak evidence of a difference in the number of RBPMS^+^ RGC after b-ITON with “pre-blast” intravitreal injections (P = 0.1364, ANOVA). Scale bar in (**D**) = 20 μm and (**E**) = 100 μm. Error bars represent mean ± SEM.

Consistent with our earlier findings at 28 dpi, there were more degenerating ON axons (arrowheads) at 14 dpi after b-ITON compared to sham controls (130 ± 26.6 vs 720 ± 116.2 axons; P < 0.0001, ANOVA, P < 0.0001 post-hoc Tukey; Fig. 4A,B). In contrast to our findings when intravitreal injection was given after b-ITON, there were fewer degenerating ON axons in mice receiving “pre-blast” siCASP2 or siEGFP intravitreal injections, with numbers similar to sham eyes, suggesting an axon-protective effect of intravitreal injections at this time point (P < 0.0001 for both compared tob-ITON without injections, post-hoc Tukey). There were fewer degenerating axons in eyes treated with siCASP2 compared to siEGFP (166 ± 22.1 axons vs 80 ± 18.23 axons; P = 0.0170, post-hoc Tukey), suggesting an inhibitory effect on axonal degeneration of caspase-2 knockdown. There was no statistical difference in the number of intact ON axons (P = 0.214, ANOVA; Fig. 4C), ON total axon density (P = 0.1099, ANOVA; Fig. 4D), or ON area between groups (P = 0.7244, ANOVA; Fig. 4E).

**Figure 5:**
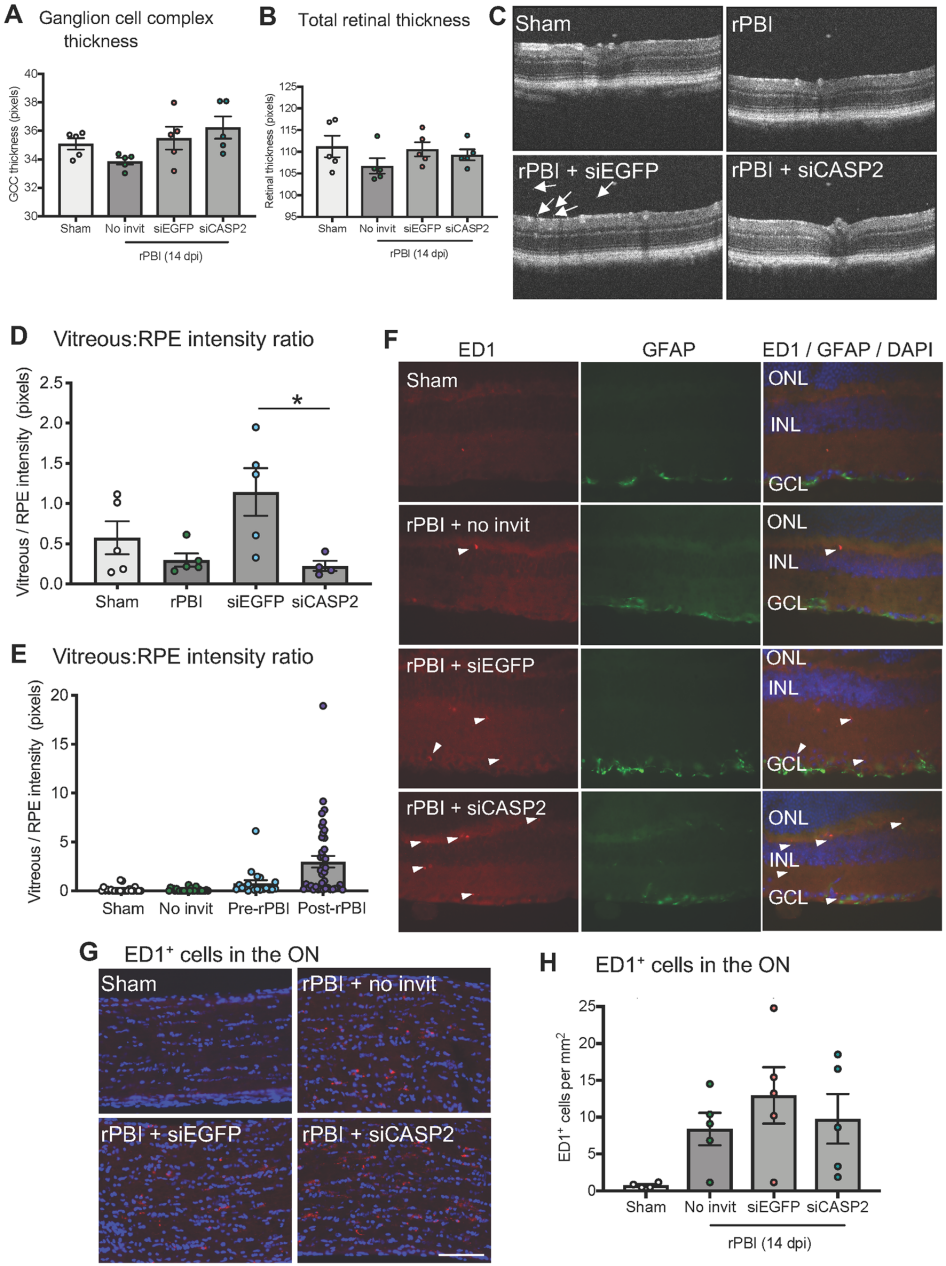
b-ITON and “pre-blast” injections had no effect on retinal thickness, but there were differential effects on vitreal inflammation. (**A**) There were no significant differences in GCC between any groups (P = 0.1016, ANOVA). (**B**) There were also no differences in total retinal thickness across any groups (P = 0.7667, ANOVA). (**C**) Representative OCT retinal scans used to quantify vitreal haze (arrows show examples of vitreous haze). (**D**) Vitreous intensity normalized to RPE intensity increased after b-ITON and siEGFP compared to b-ITON alone (P = 0.002), but did not increase with siCASP2 injections and remained similar levels to b-ITON alone (P > 0.05). There was a significant decrease in vitreous intensity between siEGFP and siCASP2 injected eyes (P = 0.033, post-hoc Tukey). (**E**) Vitreous intensity normalised to RPE intensity was also compared between grouped siCASP2 and siEGFP injections administered “pre-blast” and “post-blast”. There was greater vitreal intensity after b-ITON with “post-blast” intravitreal injections at 28 dpi compared to b-ITON with “pre-blast” intravitreal injections at 14 dpi (P = 0.001, GEE). (**F**) Representative retinal IHC images stained with DAPI (blue), ED1 (red) and GFAP (green) showing retinal ED1^+^ cell infiltration after b-ITON and greater after b-ITON with siCASP2 and siEGFP intravitreal injections. (**G**) Representative ON IHC images for DAPI (blue) and ED1 (red). (**H**) There was weak evidence for an increase in ED1^+^ cells in the ON in b-ITON groups (P = 0.075, ANOVA). Scale bars in (**F,G**) = 100 μm. Error bars represent mean ± SEM.

There was no change in RBPMS^+^ RGC counts on retinal cryosections in either injection group as compared to sham or b-ITON alone (P = 0.1364, ANOVA; Fig. 4F,G). This was further confirmed by quantification of GCC thickness on OCT scans with thickness after siCASP2 or siEGFP “pre-blast” intravitreal injections compared to eyes receiving b-ITON alone (P = 0.102, ANOVA; Fig. 5A). Moreover, retinal thickness did not change at 14 dpi after b-ITON (Fig. 5B,C, P = 0.7667, ANOVA).

### Vitreous inflammation after b-ITON and “pre-blast” intravitreal injections

OCT imaging was performed to assess vitreous inflammation by vitreous haze quantification (Fig. 5C). When b-ITON was delivered 48 h after intravitreal injection (“pre-blast” group), there was a significant increase in vitreous haze intensity between b-ITON and siEGFP-injected eyes (0.298 ± 0.08% vs 1.14 ± 0.27%, Fig. 5D) and a decrease in vitreous haze intensity 0.22 ± 0.05% in the siCASP2 injected eyes compared to siEGFP injected eyes (P = 0.0195, ANOVA, P = 0.0326, post-hoc Tukey between siEGFP vs siCASP2, Fig. 5D). Eyes which received b-ITON and siCASP2 injections were comparable to the eyes receiving no injections.

We further compared the OCT vitreal haze between b-ITON and “pre-blast” injections and “post-blast” injections, with siCASP2 and siEGFP values grouped together to determine if there was an effect of the compound injected or the injection itself. Interestingly, the vitreous intensity was lower at 2 weeks after b-ITON with “pre-blast” intravitreal injections [0.78 ± 0.28% intensity (vitreous/RPE)] compared to at 4 weeks after b-ITON with “post-blast” intravitreal injections [2.98 ± 0.059 × % intensity (vitreous/RPE)] (Fig. 5E). We also identified more inflammation in eyes injected with siCASP2, 3.20 ± 0.82% intensity (vitreous/RPE) (P < 0.001, GEE) compared to eyes injected with siEGFP, 0.78 ± 0.417% intensity (vitreous/RPE) (P = 0.063). This is consistent with our findings when b-ITON was followed by intravitreal injection (“post-blast” group; Fig. 3C).

Immunostained ED1^+^ cells in the retina were more frequently localised in the outer plexiform layer (OPL), IPL and GCL in b-ITON with siEGFP and siCASP2 injections compared to sham and b-ITON with no injections, suggesting increased inflammatory cell infiltration was caused by “pre-blast” intravitreal injections (Fig. 5F). GFAP immunostaining levels remained constant between all groups (Fig. 5F). Consistent with our findings from “post-blast” intravitreal injections, there was a trend for an increase in the number of ED1^+^ cells in the ON of mice receiving b-ITON compared to sham controls, however it did not reach statistical significance (8.40 ± 2.20 vs 0.75 ± 0.17 ED1^+^ cells per mm^2^; P = 0.075, ANOVA; Fig. 5G,H) due to the low numbers and significant variation. The number of infiltrating ED1^+^ cells were also high in b-ITON eyes injected with siEGFP and those injected with siCASP2 (12.96 ± 3.82 vs 9.77 ± 3.37 ED1^+^ cells per mm^2^; P = 0.5586 and P = 0.9742 post-hoc Tukey, both compared to b-ITON with no intravitreal injections), suggesting increased inflammatory cell infiltration was caused by blast in these groups (Fig. 5F–H). These results suggest that intravitreal injections administered 48 h before b-ITON caused less retinal and vitreous inflammation than when given 5 h after b-ITON.

## Discussion

In this study we show differential responses to intravitreal injections given 48 h before compared with 5 h after b-ITON, with the latter causing retinal and ON inflammation compared to sham controls and b-ITON alone. Receiving “post-blast” siCASP2 intravitreal injections did not affect the numbers of degenerating ON axons or the number of intact axons. In contrast, “pre-blast” siCASP2 intravitreal injections resulted in fewer degenerating ON axons compared to b-ITON with no intravitreal injections. Unexpectedly, “pre-blast” intravitreal injections of control siEGFP also resulted in similar reductions in degenerating ON as siCASP2. Also surprisingly, the extent of axon loss at 28 dpi and the extent of axon degeneration at 14 dpi were much less in this study than in other studies using this model^40,42^ which could potentially be due to the effect of the injections and the portion of the optic nerve that was examined^58^. Furthermore, there was no effect on RBPMS^+^ RGC survival after b-ITON injury with or intravitreal injections of siCASP2 or siEGFP in both “pre-blast” and “post-blast” at the time points assessed.

Compared to intravitreal injection of siEGFP, “post-blast” intravitreal injections of siCASP2 increased vitreous inflammation, assessed by OCT vitreous intensity, while siCASP2 intravitreally injected 48 h before b-ITON decreased vitreous inflammation. The hyper-reflective dots observed qualitatively were similar to previous studies which correlated these cells on OCT with histological mononuclear cells^59^, suggesting this vitreal haze could represent macrophage infiltration in response to the combination of b-ITON and intravitreal injections. These results suggest complex regulation of RGC survival and inflammation after b-ITON treated with intravitreal injections, which is dependent on timing of intravitreal injection. In addition, the intravitreal injection itself induced differential responses in b-ITON-treated mice, dependent on the timing of injection, with “pre-blast” siCASP2 reducing inflammation and “post-blast” treatment increasing inflammation. We also observed strong evidence for a reduction in ON axon density when siCASP2 was injected after the initial blast wave compared to siEGFP, and a trend towards a reduction in axon density when it was injected 48 h before, possibly due to ON gliosis known to be associated with this model^41^.

The delivery of siCASP2 by intravitreal injection at different time points around the blast wave exposures had varied effects on retinal and ON degeneration. Our first “post-blast” injection study, intravitreally injected siCASP2 or siEGFP at five hours after the initial 2 × 15 psi blast wave exposure, which was followed by two further 2 × 15 psi blast waves on consecutive days. We chose this time point to ensure that we still captured caspase-2 activation occurring < 24 h after injury allowing time for retinal siCASP2 penetration and knockdown (16 h)^16^ while remaining acceptable for clinical translation as an injured soldier or civilian may receive specialist treatment within this time frame. Notably, we observed increased vitreous inflammation when an intravitreal injection was given after the initial blast wave exposure, which was independent of the compound injected and comparable to our previous observation with necroptosis inhibitor, Necrostatin-1s^48^. There was a greater vitreous intensity detected in eyes with siCASP2 injected after b-ITON, but not in eyes receiving “pre-blast” injections compared to siEGFP control, possibly due to caspase-2 knockdown preventing apoptosis of infiltrating macrophages^60,61^, with greater macrophage infiltration and higher persistent vitreous levels of siCASP2 in the group injected after b-ITON.

In the two different treatment paradigms (injection before and after injury), the number of RBPMS^+^ RGC^62–64^ at 14 and 28 dpi were not different, suggesting that b-ITON within the 14–28 days time-frame of our experiments alone, is not enough to cause RGC death. This is perhaps not surprising as we and others detect axon degeneration prior to RGC death in models of ITON, in fact in other models the RGC death is delayed for months after injury^41,49^. For example, others have reported delayed and progressive RGC degeneration that results in reduction of GCL thickness, between 4 and 10 months after a single 26 psi blast-wave exposure^49,50^. In support of this assertion, decreased DAPI^+^ cells in the GCL was observed at 2 days after b-ITON which remained low at 28 days^41^. We have however, previously demonstrated RGC degeneration caused by intravitreal injection 5 h after b-ITON^48^ with comparable vitreous inflammation. In our current study intraocular injection after b-ITON did not affect the number of RBPMS^+^ RGC but may reflect either neuroprotection induced by both siCASP2 and off-target effects of the siEGFP or greater macrophage infiltration with neuroprotective effects. Although the same siCASP2 had little effect on RGC survival in this model, we reported site-specific caspase-2-mediated RGC death peripheral to the injury site after blunt ocular trauma but not central to the impact site, but CASP2 did not drive photoreceptor death, suggesting that caspase-2-mediated apoptosis is both cell and site specific^17,65^.

The number of degenerating ON axons was increased 28 days after b-ITON alone and was unaffected by siCASP2 and siEGFP intravitreal injections administered after b-ITON. In contrast, intravitreal injection of either siCASP2 or siEGFP given 48 h before b-ITON reduced both the number of degenerating ON axon profiles and the axon density, with a lesser effect on total axon number, suggesting some interference with the process of axonal degeneration likely due to ON gliosis associated with this model^41^. Again, despite injecting equivalent treatments, the time point of intraocular injection surrounding the blast caused different ON responses. We have previously shown degenerating ON and reduced electroretinography recordings and elevated levels of pro-inflammatory cytokines when administering an intravitreal injection at 1 day after the b-ITON, indicating that intravitreal injections may be injurious to the ON when delivered at this acute stage of ON injury^58^. The “pre-blast” study ended at 14 dpi and the “post-blast” study ended at 28 dpi, which could be viewed as a limitation of our study, but we did not intend to compare the two protocols to each other. These timepoints of analysis were chosen since we previously showed that 2 weeks was the peak of axon degeneration in this model and at 4 weeks significant axon loss was detected^41^. Thus, the 2-week time point was used as the most robust anatomical assessment of protection by quantification of axon degenerative profiles, whilst at the 4-week time point the most robust anatomical assessment of protection was quantification of total axons.

Long-lasting morphological and functional consequences in the eye have also been observed in models of repetitive mild traumatic brain injury (r-mTBI)^66^. For example, r-mTBI in a mouse model caused a decrease in ON diameters, increased cellularity and areas of demyelination in the ON. This was consistent with areas of decreased cellularity in the GCL and 67% reduction in Brn3a^+^ RGC. Furthermore, SD-OCT demonstrated thinning of the inner retina whilst ERG demonstrated a decrease in the amplitude of the photopic negative response without changes in a- or b-wave amplitudes. In a separate single blast TBI model, the authors also found decreases in RNFL thickness and reduced cellularity in the GCL at 3-months with accompanying changes in retinal function^50^. However, r-mTBI led to more profound and widespread damage to the RNFL. These studies suggest that visual system dysfunction might be a common feature after blast and repeated blunt mTBI.

As we have previously reported^48^, there were infiltrating ED1^+^ cells in the ON at 28 days after b-ITON alone and in eyes receiving pre-blast and post-blast injections and have now shown infiltrating ED1^+^ cells at 14 days. ED1^+^ cells are likely to be infiltrating inflammatory macrophages to clear myelin debris from degenerating ON axons^67^, and would be consistent with previous findings of CD68^+^ cells infiltrating the brain after blast injury^68^, and macrophage ON infiltration after ultrasonic injury^69^. However, macrophages recruited into the ON may exert polarised effects since they are not only toxic to neurons and glia but can also promote CNS axon regeneration^70^. Vitreal inflammation, induced by lens injury or injection of zymosan has long been known to cause release of oncomodulin, promoting RGC neuroprotection and axon regeneration^71–74^. In this study, RGC and ON degeneration may reflect a complex balance between pro-degenerative ON macrophage infiltration and neuroprotective retinal and vitreous macrophage infiltration, with more neurodegeneration in the “pre-blast” injection group, which displayed less vitreous inflammation and neurodegeneration, than the “post-blast” injection group which had more vitreous inflammation.

Intravitreal drug delivery has become routine for the delivery of drugs, suspensions and intraocular implants into the vitreous cavity. It is the main route to deliver macromolecules to the posterior segment of the eye. Although the technique leads to targeted delivery of therapeutics, it is invasive since it requires the penetration of the globe and is associated with complications such as endophthalmitis, retinal detachment, cataracts and intraocular haemorrhage^75^. Intraocular injections can be uncomfortable, may have limited patient compliance and often require multiple injections which can increase the risk of side effects such as infectious endophthalmitis and retinal detachment^76^. However, repeated intravitreal injections of anti-angiogenic agents such as vascular endothelial growth factor (VEGF) inhibitors have become the first-line treatment for exudative age-related macular degeneration (AMD)^77^. Our study which detected additive effects of combined b-ITON and intraocular injections^48^, suggests that intraocular injection may not be the optimal therapeutic delivery method for treating ocular blast injury and that this should be considered in development of treatments for humans. However, caution needs to be exercised when projecting adverse effects of intraocular injections in mouse eyes to substantially larger human eyes since an intraocular injection in a mouse eye may inflict a greater degree of injection-related ocular injury than in the larger human eye with a ~ 1000-fold larger vitreous volume^78,79^. Hence, the overall effects of intraocular injections into the human eye may be negligible and needs further investigation.

In conclusion, despite evidence of caspase-2 activation after b-ITON, we did not detect a neuroprotective effect of caspase-2 knockdown in this model, possibly because of the limited loss of RGC soma. We demonstrate that intravitreal injection and b-ITON combined cause retinal and vitreal inflammation, with a greater effect when the injection was administered after b-ITON compared to before the injury. Depending on the timing of injection, intravitreal injection may also induce RGC axonal loss, although this was minimal. Similarly, the timing of the intravitreal injections with respect to b-ITON also determined whether siCASP2 reduced or increased vitreous inflammation. The time point of intravitreal injection surrounding ocular trauma can induce varied retinal and ON pathologies and should be carefully considered when testing treatments in models of ocular trauma and in treating ocular trauma patients.

## Supplementary Information

Supplementary Information 1.

Supplementary Information 2.

## Data Availability

All data are presented either in the main text or in the data supplement. The datasets generated and/or analyzed during the current study are available from the corresponding author on reasonable request.
