# Supplementary material for: Effects of intravitreal injection of siRNA against caspase-2 on retinal and optic nerve degeneration in air blast induced ocular trauma
Source: Sci Rep. 2021 Aug 19;11:16839. doi: 10.1038/s41598-021-96107-y (PMC8377143; doi:10.1038/s41598-021-96107-y)
Supplement: Supplementary file 1 — Supplementary Information 1. [file 41598_2021_96107_MOESM1_ESM.docx]

**Supplementary Information**

**Effects of intravitreal injection of siRNA against caspase-2 on retinal and optic nerve degeneration in air blast induced ocular trauma**

Chloe N Thomas ^1, 2^, Alexandra Bernardo-Colón ^3^, Ella Courtie ^1, 4^, Gareth Essex ^1^, Tonia S Rex ^3, 5^, Richard J Blanch ^1, 4, 6, †^*, Zubair Ahmed ^1, †^*

^1^Neuroscience and Ophthalmology, Institute of Inflammation and Ageing, College of Medical and Dental Sciences, University of Birmingham, Birmingham, UK.

^2^School of Biomedical Sciences, Institute of Clinical Sciences, College of Medical and Dental Sciences, University of Birmingham, Birmingham, UK.

^3^Vanderbilt Eye Institute, Vanderbilt University Medical Center, Nashville, TN, USA.

^4^Ophthalmology Department, University Hospitals Birmingham NHS Foundation Trust, Birmingham, UK

^5^Department of Ophthalmology and Visual Sciences, Vanderbilt University School of Medicine, Nashville, TN, USA.

^6^Academic Department of Military Surgery and Trauma, Royal Centre for Defence Medicine, Birmingham, UK.

^†^RJB and ZA are joint senior and corresponding authors.

***Correspondence:** RJB ([r.j.blanch@bham.ac.uk](mailto:r.j.blanch@bham.ac.uk)) or ZA ([z.ahmed.1@bham.ac.uk](mailto:z.ahmed.1@bham.ac.uk)). Tel: +44 (0) 121 414 8859 (RJB and ZA).

**Figure Legends**

**Supplementary Figure 1.** **Assessment of RGC survival.** (**A**) Schema for quantification of RGC numbers from retinal wholemounts. (**B**) Schema for quantification of RGC numbers from cryosections stained with RBPMS. ON = optic nerve; ONH = optic nerve head, RGC = retinal ganglion cells.
