# Supplementary figures and images for: Effects of intravitreal injection of siRNA against caspase-2 on retinal and optic nerve degeneration in air blast induced ocular trauma
Source: Sci Rep. 2021 Aug 19;11:16839. doi: 10.1038/s41598-021-96107-y (PMC8377143; doi:10.1038/s41598-021-96107-y)

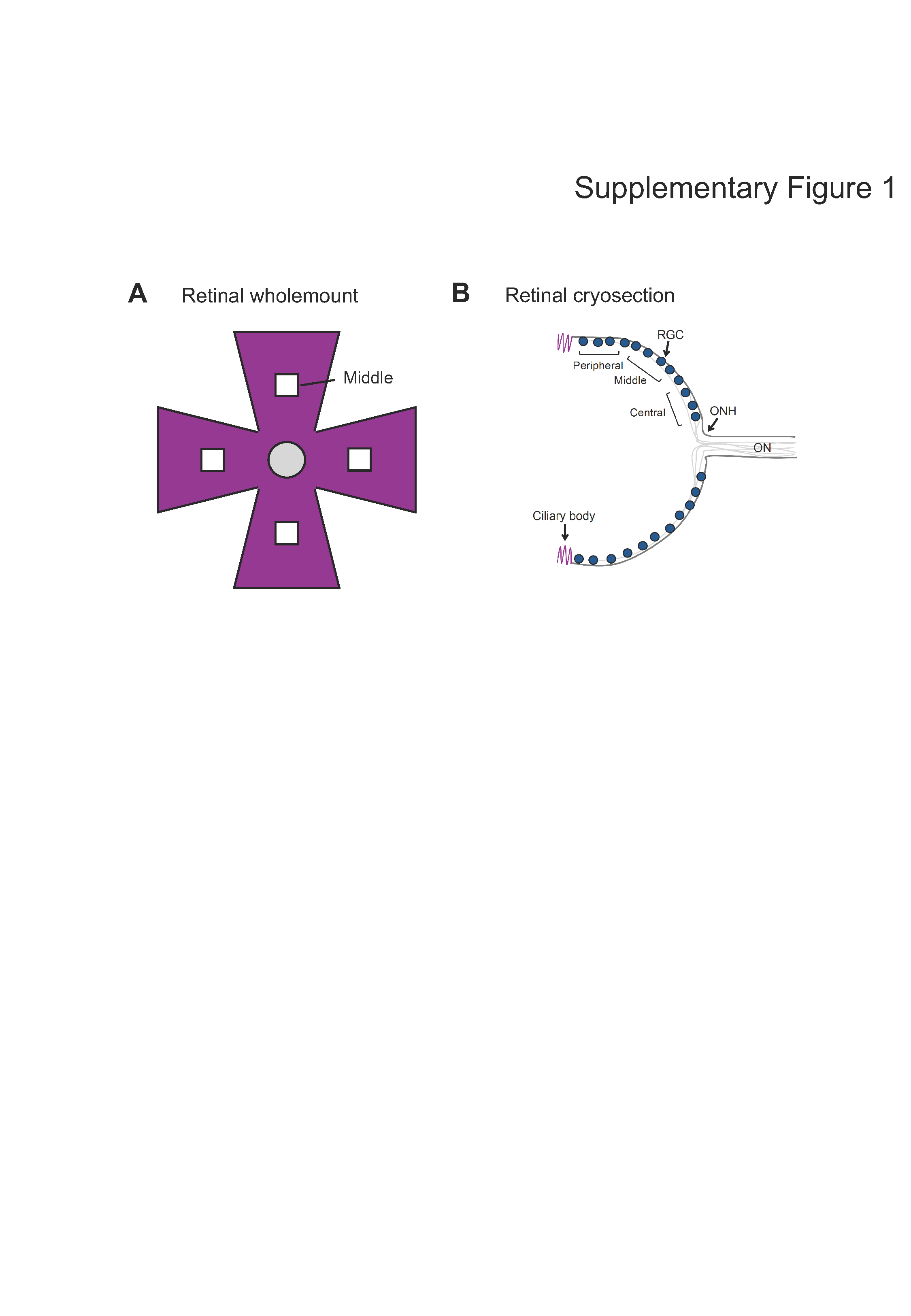

Supplement: Supplementary file 2 — Supplementary Information 2. [file 41598_2021_96107_MOESM2_ESM.tiff]
